# Cyclic Economy-Driven Composites for Material Extrusion Three-Dimensional Printing: Poly(methyl methacrylate) from Recycled Scrap with Optimized Biomass-Derived Biochar Filler Content

**DOI:** 10.1021/acsomega.5c02525

**Published:** 2025-06-06

**Authors:** Nectarios Vidakis, Nikolaos Michailidis, Dimitrios Kalderis, Emmanuel Maravelakis, Vassilis Papadakis, Apostolos Argyros, Nikolaos Mountakis, Maria Spyridaki, Nektarios Nasikas, Markos Petousis

**Affiliations:** a Department of Mechanical Engineering, 112178 Hellenic Mediterranean University, Heraklion 71410, Greece; b Physical Metallurgy Laboratory, Mechanical Engineering Department, School of Engineering, Aristotle University of Thessaloniki, 54124 Thessaloniki, Greece; c Centre for Research & Development of Advanced Materials (CERDAM), Center for Interdisciplinary Research and Innovation, Balkan Centre, Building B’, 10th km Thessaloniki-Thermi road, Thessaloniki 57001, Greece; d Department of Electronic Engineering, 112178 Hellenic Mediterranean University, Chania 73133, Greece; e Department of Industrial Design and Production Engineering, University of West Attica, Athens 122 43, Greece; f Institute of Electronic Structure and Laser, Foundation for Research and Technology−Hellas, N. Plastira 100m, Heraklion 70013, Greece; g Division of Mathematics and Engineering Sciences, Department of Military Sciences, 69139 Hellenic Army Academy, Vari, Attica 16673, Greece

## Abstract

Environmentally friendly materials are emerging materials that find applications in an increasing number of cases when being three-dimensional printed (3D-P), as they can provide many possibilities and offer unique properties to fulfill industrial needs and requirements. As part of that effort, recycled (from sheet trimmings waste) poly­(methyl methacrylate) (PMMA) and (nature-sourced) biochar were selected to be combined and examined herein. Composites with Biochar concentration in the 0.0–10.0 wt % (step 2.0 wt %) range were assessed. Compounds were extruded into filaments, which fabricated coupons (material extrusion 3D-P) for the tests that followed. The samples were experimentally evaluated for their characteristics related to mechanical, chemical, rheological, and thermal behavior, as well as for their structure and morphology. Mechanical testing included tensile, bending, and Charpy Notched coupons’ investigation. The microhardness was also measured. In addition, quality characteristics were assessed through porosity and dimensional deviation data analysis. The composite distinguished in relation to pure PMMA was PMMA/Biochar 6.0 wt % (>20% increase in the strength on the tensile and flexural experiment), as most of the investigated properties revealed their greatest values in that Biochar concentration. Furthermore, the eco-friendly biochar addition positively affected most of the recycled PMMA characterization metrics assessed, showing potential for the environmentally friendly composites developed herein for the 3D-P process.

## Graphical abstract

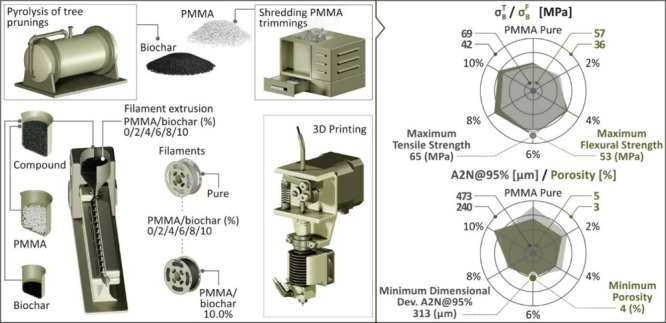

## 1 Introduction

The material extrusion (MEX) method is part of the additive manufacturing (AM) family of technologies (commonly referred to as three-dimensional (3D) printing – 3D-P) and is based on the deposition of selected (polymeric) materials through a heated nozzle of a 3D printer, following the shape, structure, and dimensions that have been previously designed.^1^ Applications of AM include biomedical,^2–5^ food,^6–8^ construction and architecture,^9,10^ aerospace,^11^ electronics,^12,13^ etc. The field of 3D printing (3D-P) has become increasingly familiar with biomaterials, and new application prospects have been created, providing them with the ability to receive products with improved and enhanced properties. One way to approach biomaterials is to combine polymers as matrix materials with bioderived fillers. Many polymers have been employed in AM 3D printing, including Polylactic Acid (PLA),^14^ polycaprolactone (PCL),^15^ polypropylene (PP),^16^ Acrylonitrile Butadiene Styrene (ABS),^17^ polyamide (commercially known as nylon) 6 (PA6),^18^ High-density polyethylene (HDPE),^19,20^ and poly­(Methyl Methacrylate) (PMMA).^21^ Their ability to withstand reprocessing and be reused in MEX AM has attracted the research community’s interest, with the reports showing potential for reusing after six successive thermomechanical procedures.^22,23^

PMMA, also known by the commercial name “plexiglass”, is a thermoplastic material (optically clear amorphous) created from the methyl methacrylate monomer when conducting free radical vinyl polymerization.^24^ It possesses unique characteristics, including low density, cost-effectiveness, manipulation ease, adjustable physical and mechanical properties,^25,26^ and optical and insulating qualities.^27^ As a result, biomaterials, along with other polymers,^28,29^ are suitable for applications related to dental issues^30,31^ as well as catalysis, sensors, solar cells, photodetectors,^32^ and various industrial applications.^33^ The mechanical properties of PMMA have been reported to be modified in various studies, focusing on the bending strength,^34–38^ impact strength,^39,40^ fracture toughness,^41,42^ and surface hardness.^43^ Literature reports that it can be welded with the friction stir welding (FSW) method and even hybrid joints of PMMA sheets and 3D printed parts can be achieved, facilitating its use in respective applications.^44^ In AM, its sustainability has been proven, as it was found that it can be thermomechanically reprocessed up to six times without notable degradation of its mechanical and other properties.^45^ Its mechanical properties have also been investigated^46^ and optimized with regard to the required energy for the fabrication of items utilizing AM (contributing to sustainability and efficiency).^47^ Furthermore, it has been utilized before as a matrix material for the preparation of multifunctional compounds with enhanced mechanical properties and antibacterial performance.^48^

After researching the PMMA market size, valuable indicative information was derived regarding the upcoming growth of PMMA. Mordor Intelligence reported a 2024–2029 market size prediction, stating a Compound Annual Growth Rate (CAGR) of 6.57%, from US dollar 9,160 million (2024) to US dollar 12,590 million (2029).^49^ Grand View Research examined the global PMMA market size in the 2023–2030 forecast period and indicated that US dollar 5,419.3 million in 2022, are about to meet growth of 5.3% CAGR between 2023 and 2030.^50^ An additional report possessed by Fact.MR indicated a 5.9% CAGR between 2022 and 2032 from US dollar 4,900 million to US dollar 8,500 million.^51^ Then for the forecast period 2024–2034, Precedence Research revealed that US dollar 5,430 million in 2024 is expected to reach US dollar 9,280 million in 2034 (5.5% CAGR).^52^ The global production of PMMA is approximately 3.9 million metric tons annually.^53^ However, only about 10% of this amount is recycled each year, resulting in the remaining 90%, or approximately 3.5 million metric tons, being either landfilled, incinerated, or released into the environment.^54^ Globally, the generation of PMMA waste is estimated to be between 800,000 and 1,000,000 t per year, which corresponds to 20–25% of Methyl methacrylate usage. Despite the high recycling value of PMMA, current processes lead to the majority of PMMA waste being disposed of in landfills or incinerated, underscoring the necessity for improved recycling methods and facilities.

Biochar is produced by following a specific high-heating procedure based on the thermomechanical conversion of biomass with limited oxygen.^55,56^ The renewable organic material of biomass is basically an agricultural product, as it can be created with the assistance of animals and plants.^57^ In particular, some biochar sources are animal manure,^58–60^ crop straw,^61,62^ sewage sludge,^63–65^ wood residues,^66,67^ or even food waste.^68–70^ Biochar is characterized by its stability and high porosity,^71^ and is distinguished by its cost-effectiveness, eco-friendliness, and reusability.^72,73^ It is utilized for applications related to agriculture,^74^ water treatment,^75–77^ medicine,^78^ and building materials.^79^ There are various biochar production technologies, namely fast or slow pyrolysis, gasification, and torrefaction,^80^ but several process parameters prevail.^81^

With regard to the size of the biochar market, Grand View Research reported 2024–2030 13.9% CAGR growth from the initial US dollar 541.8 million market size in 2023.^82^ Globe Newswire indicated an 8.9% CAGR between 2024 and 2033, reaching US dollar 136.2 million in 2033, from US dollar 58.3 million in 2023.^83^ In another report,^84^ it was reported US dollars will be 253.29 million in 2023, to be projected at about US dollar 622.8 million by 2030 (14.0% CAGR). Moreover, based on the indicated information by Fortune Business Insights, the US dollar 763.48 million global Biochar market size in 2024 is going to increase from US dollar 859.04 million in 2025 to US dollar 2,097.72 million in 2032 (CAGR 13.6%).^85^ The International Biochar Initiative (IBI) and the US Biochar Initiative (USBI) estimated the global biochar production to be in 2023 a minimum of 350,000 t annually.^86,87^ This marks a substantial increase from previous years, with a compound annual growth rate (CAGR) of 91% from 2021 to 2023. Projections for the future indicate that by 2050, biochar production could range from approximately 63 to 118 million metric tons annually, contingent upon various adoption scenarios.^88,89^

Biochar has been part of some research work in existing literature related to its composition with different polymeric matrix materials^90,91^ elaborated in the 3D printing field as well.^92^ For example, Polylactic acid (PLA)/ Biochar composites were subjected to fused deposition modeling and investigation of their mechanical behavior, considering the filler content and 3D printing parameters.^93–95^ Polypropylene (PP)/ Biochar composites also achieved reinforced properties.^96,97^ Biochar has also been combined with recycled poly­(ethylene terephthalate glycol) (PETG) and poly­(ethylene terephthalate) (PET), proven to be effective in improving the properties of the samples.^98,99^ Other polymeric matrices utilized for biochar-based composites development include high-density polyethylene (HDPE),^100^ and acrylonitrile butadiene styrene (ABS).^101^ Each filler interacts with each polymeric matrix in a different way, therefore individual studies are required for each combination and composites preparation method,^102,103^ justifying the need for such endeavors. In the existing literature, biochar has proven a strong potential as an eco-friendly reinforcement agent for polymeric matrices in the MEX method, while it improved the quality characteristics of the 3D prints as well.^104^ However, the literature seems to lack investigations of 3D printed composites with PMMA materials other than resins,^105,106^ which constitutes a gap in the research field. The rationale herein was to enhance the PMMA with a sustainable filler, such as biochar, to introduce new compounds for MEX AM with improved performance for the applications PMMA is used. These applications as stated in the manuscript include catalysis, sensors, solar cells, photodetectors, and various industrial applications. It should be noted that PMMA is popular in optical applications, which is an area that cannot be applied as a PMMA/biochar compound since the addition of biochar darkens the color of the compounds and removes their transparency.

Herein, PMMA/Biochar mixtures of 0.0–10.0 wt % (0.0 wt % was the pure PMMA, as the control sample) filler concentrations were created followed by filaments, and then different types of coupons. The composite samples were investigated thermally, rheologically, mechanically, structurally, and morphologically by conducting a series of tests suitable for deriving the desired information. The selection of such fillers is due to the attempt to achieve and promote biobased and eco-friendly products manufactured by industries, as well as to support the bioeconomy. Initially, the raw materials were measured and mixed before being supplied to the filament extrusion apparatus. Then, the filaments were employed for the 3D printing of coupons.

Examples from all of the PMMA composite samples were assessed for their tensile and bending characteristics, namely strength (σ_B_^T^, σ_B_^F^), modulus of elasticity (E^T^, E^F^), impact strength (Charpy) (T_IT_^C^), microhardness (M-H), and tensile toughness (T^T^). The filaments were subjected to tensile and bending tests. In addition to the mechanical behavior, the compounds were thermally assessed by thermogravimetric analysis (TGA) and differential scanning calorimetry (DSC), rheologically tested by material flow rate (MFR) and viscosity, structurally investigated, and morphologically inspected by scanning electron microscopy (SEM). The effect of biochar on quality metrics, such as the geometrical accuracy of the prints and their porosity (number of voids in the structure formed by the 3D printing process), were investigated with microcomputed tomography (μ-CT). As the authors’ research revealed, PMMA has not yet been combined with biochar and investigated on 3D-printed samples. Consequently, this study provided unique and valuable information and data. In this case (PMMA matrix), biochar proved its capability to be used as an eco-friendly alternative to common metal fillers.

## 2 Materials and Methods

### 2.1 Materials

Plazcryl PMMA from Plazit Polygal Inc. (Charlotte, North Carolina, United States) granules were obtained by shredding 4 mm thick PMMA sheet trimmings, which later underwent washing and drying in order to be clear, ready, and suitable for extrusion into filaments. The datasheet provided by the supplier stated that some of its characteristics are namely density of 1.19 g/cm^3^, σ_*B*_^*T*^ of 72 MPa, σ_*B*_^*F*^ of 106 MPa and Charpy impact strength of 15 kJ/m^2^.

The filler selected for this work was biochar from prunings derived from olive trees in Chania, Crete, Greece, after following a specific procedure. The first step consisted of washing and clearing the impurities of the biomass, followed by air-drying, before flame-curtain pyrolysis. The remaining steps have already been presented in previous research work,^9^ and additional details and necessary specifications are provided in the literature.^107^ For the pyrolysis, the prunings were systematically divided into three groups, and flame-curtain pyrolysis was conducted thrice for each group. Each pyrolysis session was maintained for 1 h at a temperature of 540 ± 50 °C. As documented in the literature, temperature variations in this type of reactor can be substantial.^108^ To mitigate this, temperature was monitored using four thermocouples positioned at various points on the kiln’s outer surface. During the initial phase of establishing the flame cap, significant temperature fluctuations were observed. However, once the feeding rate of prunings (layering process) was stabilized, the temperature also stabilized. In flame cap pyrolysis, maintaining uniform feedstock and an optimal feeding rate is crucial to minimizing temperature fluctuations. Once the first batch of prunings began to pyrolyze, no external heat source was required, rendering the process self-sustaining. Following quenching with water, each batch of biochar was air-dried for 96 h and weighed. The three samples were then amalgamated into a single composite sample and homogenized. The sample was ground using a Sepor-type rod mill. After thorough sieving, the fraction smaller than 100 μm was stored for further analysis and use. The distribution of biochar particle size was determined using a Malvern type-S Mastersizer (Malvern Instr., Malvern, UK) with laser diffraction. Elemental analysis (C, H, N, S) was conducted using a ThermoFlash-2000 combustion analyzer (ThermoFisher Sc., UK). The ash content was determined following the European Biochar Certificate (EBC) Guidelines (EBC-2012, ver. 9.3E). The oxygen content was calculated by difference. The chemical composition of the sample was analyzed using an X-ray fluorescence (XRF) spectrometer (Rigaku ZSX PrimuII, Japan). Biochar was examined with Scanning Electron Microscopy to reveal the shape and size of the produced particles. Images are provided in Supporting Information.

### 2.2 Composite Preparation, Filament Extrusion, and 3D-Printed Coupon Manufacturing

Six composites (0.0, 2.0, 4.0, 6.0, 8.0, 10.0 wt %) were aimed at being produced for this work, with filler concentrations being selected after conducting tests on fabricated samples and discovering up to which filler quantity the behavior of the samples was not improved anymore (saturation phenomena.^109,110^ The materials were prepared by measuring the desired amounts to create each composite and then transporting them into a laboratory blender operating at high wattage (400W, 4000 rpm, 20 min). Filament extrusion was supplied later by the produced composites, derived after the mixing procedure, and was executed by a model named Precision 450 from the company 3D Evo B.V, established in Utrecht, The Netherlands, producing 1.75 mm filaments (3D printing acceptable). Coupon manufacturing was performed using an apparatus named Funmat HT from the company Intamsys Technology Co. Ltd., established in the city of Shanghai, in China. The coupons were fabricated to be compatible with four different tests (tensile, bending, and Charpy), creating a total of four coupon types. In the Supporting Information, Figure S1, and Figure S2 show filament images, as well as the results from their diameter monitoring and mechanical testing. Moreover, in Figure S3, the 3D printing settings are utilized, along with the designs and dimensions of the created tensile, bending, and Charpy Notched coupons.

### 2.3 Raman, Rheological, and Thermal Characterization

To acquire spectra with the Raman method, a LabRAM HR apparatus, by the Horiba Scientific company, established in the city of Kyoto, Japan, was used. The rheological investigation was based on viscosity and MFR tests (ASTM D1238–13), and the data were extracted from the rheometer results. These methods along with TGA and DSC are presented in the supplementary file.

### 2.4 Mechanical Evaluation

Mechanical characterization was conducted using four different types of tests: tensile, bending, Charpy notched impact, and M-H. The same device was used for the tensile (ASTM D638–14, 3.2 mm thickness, type V) and bending (ASTM D790–10) tests, namely, an Imada MX2 motorized test stand from Imada Inc. (Northbrook, Illinois, USA), each equipped with the corresponding grips. Charpy notched impact information (ASTM D6110) was obtained employing a Terco MT220 instrument from Terco AB (Kungens, Sweden). M-H (Vickers) (ASTM E384–17) tests were conducted with the assistance of the apparatus Test 300 from the company Innovatest Europe BV, established in the city of Maastricht, The Netherlands. In all tests, five samples were evaluated in accordance with the requirements of the respective standards followed. Experiments were conducted in ambient room conditions (23 °C and 55% humidity).

### 2.5 Morphology and Structure Examination

SEM was performed on the vertical and fracture sections of the coupons to investigate their morphology, using an apparatus model named JSM-IT700HR, which is a field emission SEM from the company Jeol Ltd., established in the city of Tokyo, Japan, with images captured at different magnifications. The structural characteristics of the samples, namely their porosity and dimensional deviation, were assessed by microcomputed tomography (μ-CT) using a Tomoscope HV Compact from Werth-Messtechnik GmbH (Germany). These structural characteristics are directly related to the print quality of the coupons considering that the PMMA matrix is recycled and the (eco-friendly) biochar filler affects these aspects.

## 3 Results

### 3.1 Raman Evaluation

Figure 1 depicts the profiles derived with Raman spectroscopy of the unfilled PMMA and PMMA compounds. Additionally, the related Raman peaks from the unfilled PMMA are depicted in a table in the supplementary file. Values were extracted from the respective bibliography together with their reference.

**Figure 1:**
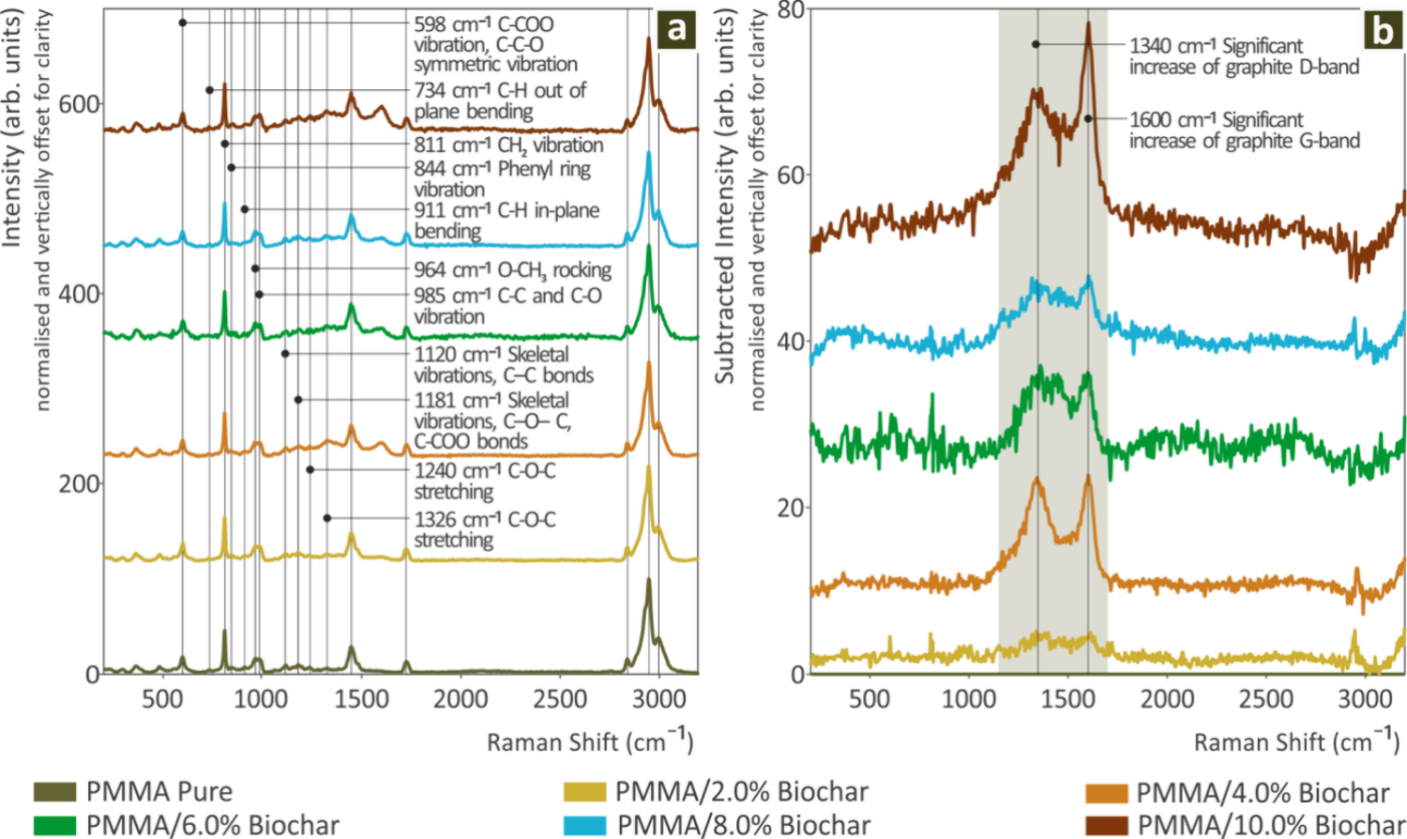
(a) Raman spectra from PMMA Pure, PMMA/Biochar 2 wt %, PMMA/Biochar 4 wt %, PMMA/Biochar 6 wt %, PMMA/Biochar 8 wt %, and PMMA/Biochar 10 wt %, and (b) Raman spectral differences of PMMA/Biochar 2 wt %, PMMA/Biochar 4 wt %, PMMA/Biochar 6 wt %, PMMA/Biochar 8 wt %, and PMMA/Biochar 10 wt % from PMMA Pure.

As can be seen in Figure 1b, the addition of Biochar in PMMA presented a gradual intensity increase in the two graphite bands D-band at 1340 cm^–1^ and G-band at 1590 cm^–1^ ^111,112^ which are commonly found in Biochar, in all Raman spectra from the PMMA samples, as shown in Table 1.

**Table 1:** PMMA/Biochar and PMMA pure Differences in the (Significant) Raman Peak

1340	gradual increase	significant increase in graphite D-band
1600	gradual increase	significant increase in graphite G-band

### 3.2 Rheological and Thermal Characterization

Figure 2a shows viscosity and stress profiles versus shear rate (240 °C) of PMMA/Biochar 0.0–10.0 wt %, revealing the increase of stress as the viscosity decreases. The rise of biochar content in the compounds decreased the viscosity (pseudoplastic behavior) of the compounds. A nonmonotonic behavior is observed, with an initial decrease on the 2 wt % compound, then higher viscosity is depicted for the 4 and 6 wt % compounds and then the 8 wt % compound has a similar response to the 2 wt % compound, while in the 10 wt % viscosity is further decreased. This interesting finding is analyzed further below in the discussion section. Figure 2b shows bars indicating the MFR levels of the same composite samples (230 °C). The highest MFR value was detected for the unfilled PMMA, as the addition and increase of the filler resulted in reduced MFR levels (poorer flowability). Compounds from 2 to 8 wt % biochar content exhibited similar MFR values, while in the highest loaded compound (10 wt %) the MFR value was further decreased.

**Figure 2:**
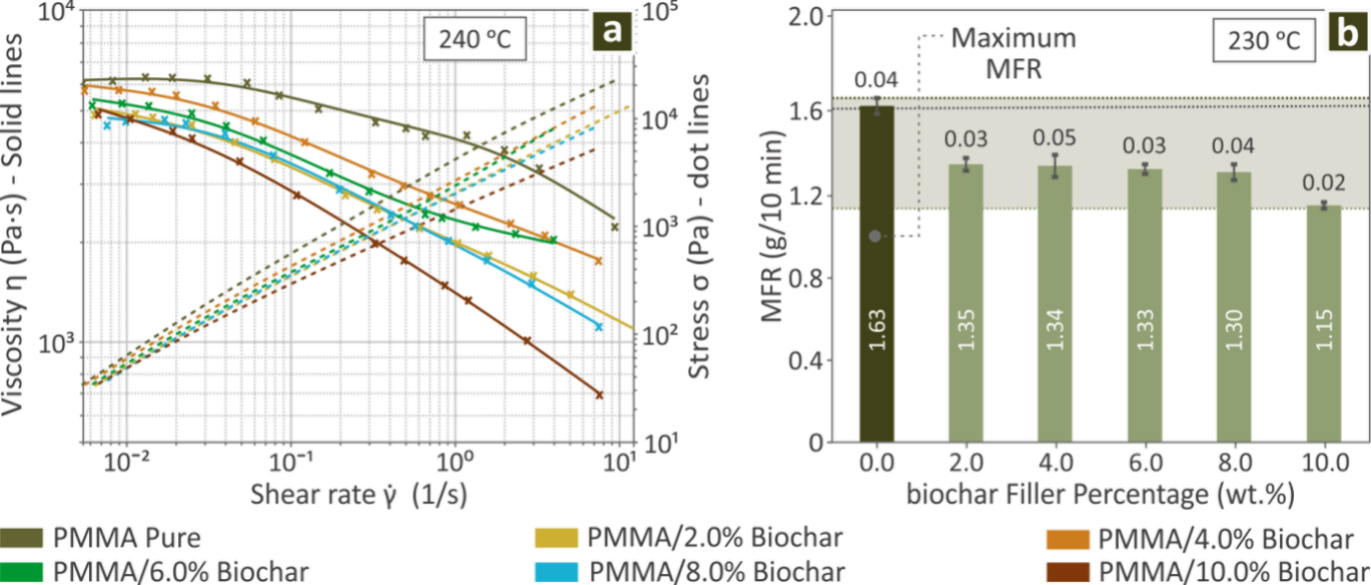
(a) Viscosity and stress profiles versus the shear rate of PMMA/Biochar (0.0–10.0 wt %) and (b) bars revealing the MFR levels of PMMA/Biochar (0.0–10.0 wt %).

Figure 3 presents the test results related to the thermal behavior of the PMMA/Biochar (0.0–10.0 wt %) composites. Figure 3a depicts the weight versus temperature profiles derived from TGA, revealing that the additive content increase leads to lesser weight loss. Figure 3b depicts the heat flow compared to temperature results from the DSC, while Figure 3c and d show the glass transition temperature (*T*_g_) levels detected during DSC, as well as the initial decomposition temperature (IDT) and final residue (FR) levels detected during TGA, respectively. It was observed that the maximum level for IDT was detected in pure PMMA, while FR increased as expected with the rise of the filler percentage.

**Figure 3:**
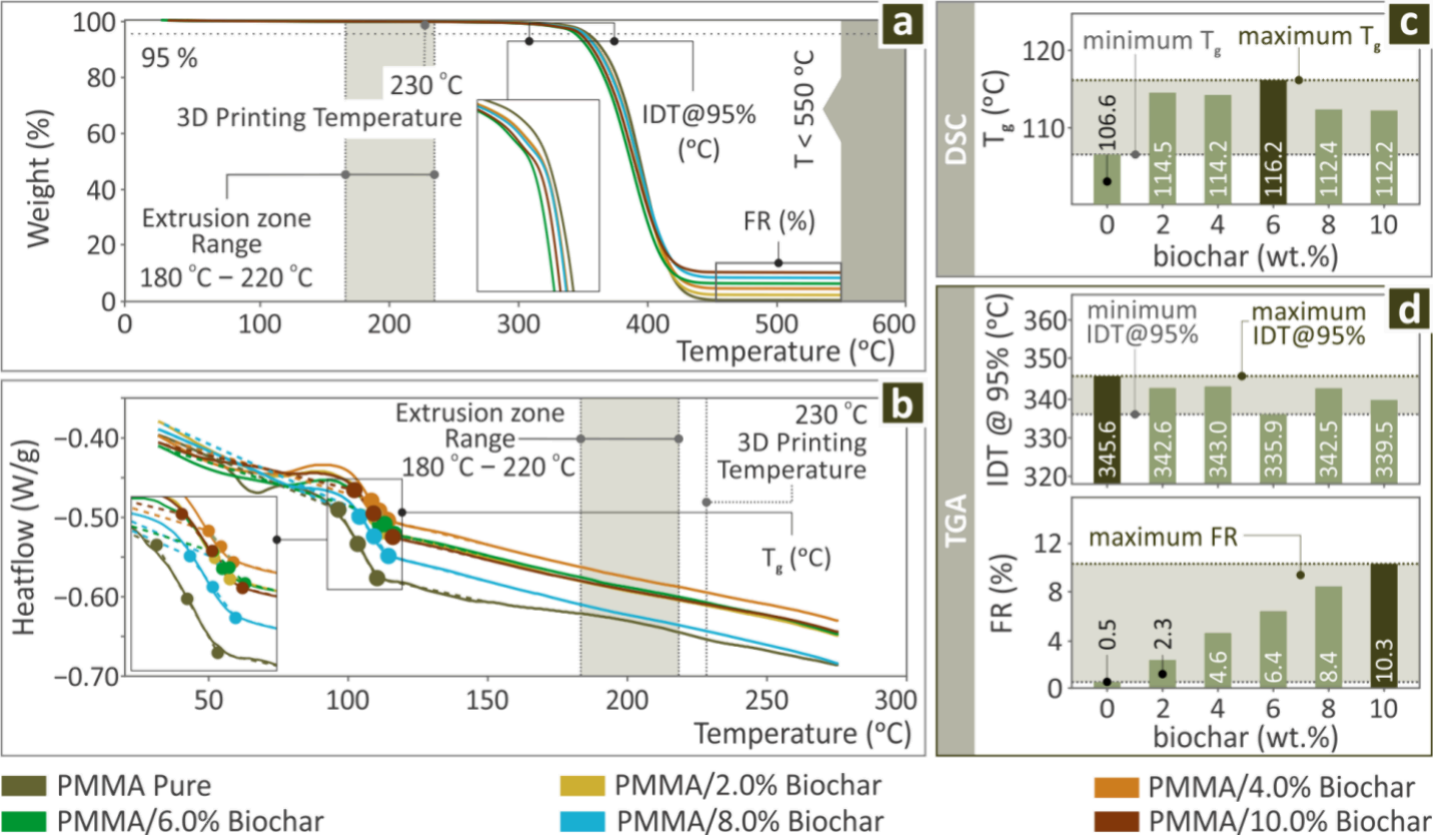
For PMMA/Biochar (0.0–10.0 wt %) samples (a) TGA measurements and (b) DSC profiles, (c) *T*_m_, and (d) IDT and FR levels in bars.

### 3.3. Tensile, Bending, Charpy Impact, and M-H Results

Figure 4 shows the bars revealing the tensile properties of the Biochar (0.0–10.0, wt %) coupons, namely σ_B_^T^ (Figure 4a), E^T^ (Figure 4b) and T^T^ (Figure 4c). PMMA/Biochar 6.0 wt % is distinguished for its σ_B_^T^ and T^T^ performance with an improvement of 21.9% and 20.2% over pure PMMA, while PMMA/Biochar 8.0 wt % for its E^T^ levels, by being 20.5% enhanced over pure PMMA. In Figure 4a, there is also an image captured during tensile testing, while in Figure 4b and c, there are pictures of two failed coupons, one belonging to the 8.0 wt % and one to 6.0 wt % biochar content compounds.

**Figure 4:**
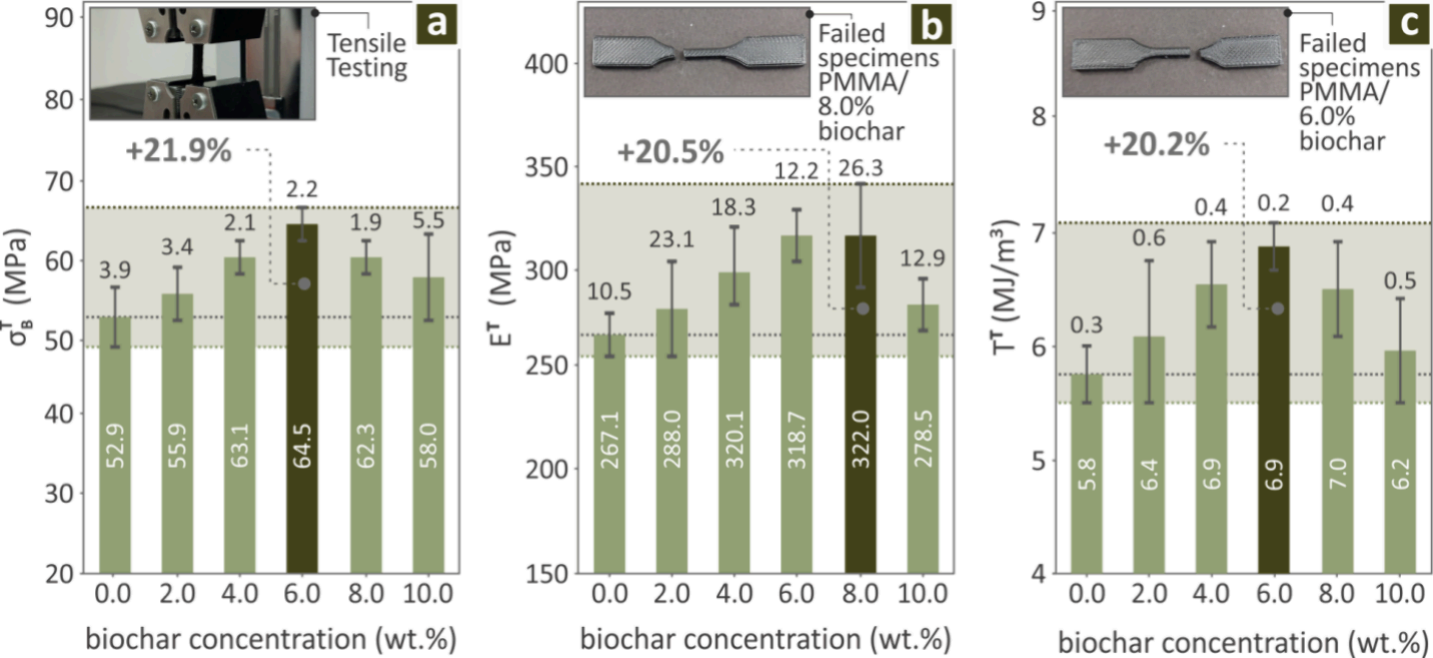
For PMMA/Biochar (0.0–10.0 wt %) tensile tested coupons, results with regard to (a) σ_*B*_^*T*^ (b) *E* ^*T*^, and (c) *T* ^*T*^, along with images depicting the tensile testing procedure, one PMMA/Biochar 8.0 wt %, and one to PMMA/Biochar 6.0 wt % failed coupons, respectively.

Figure 5 presents the bars of the bending property levels for PMMA/Biochar (0.0–10.0, wt %) coupons, namely, σ_B_^F^ (Figure 4a), E^F^(Figure 4b). Again, the PMMA/Biochar 6.0 wt % revealed a great performance in both of the two properties, being 20.1% (σ_B_^F^) and 20.3% (E^F^) enhanced over pure PMMA. In addition, Figure 5a shows an image from a coupon bending test, while Figure 5b includes an image of a Biochar 6.0 wt % failed specimen.

**Figure 5:**
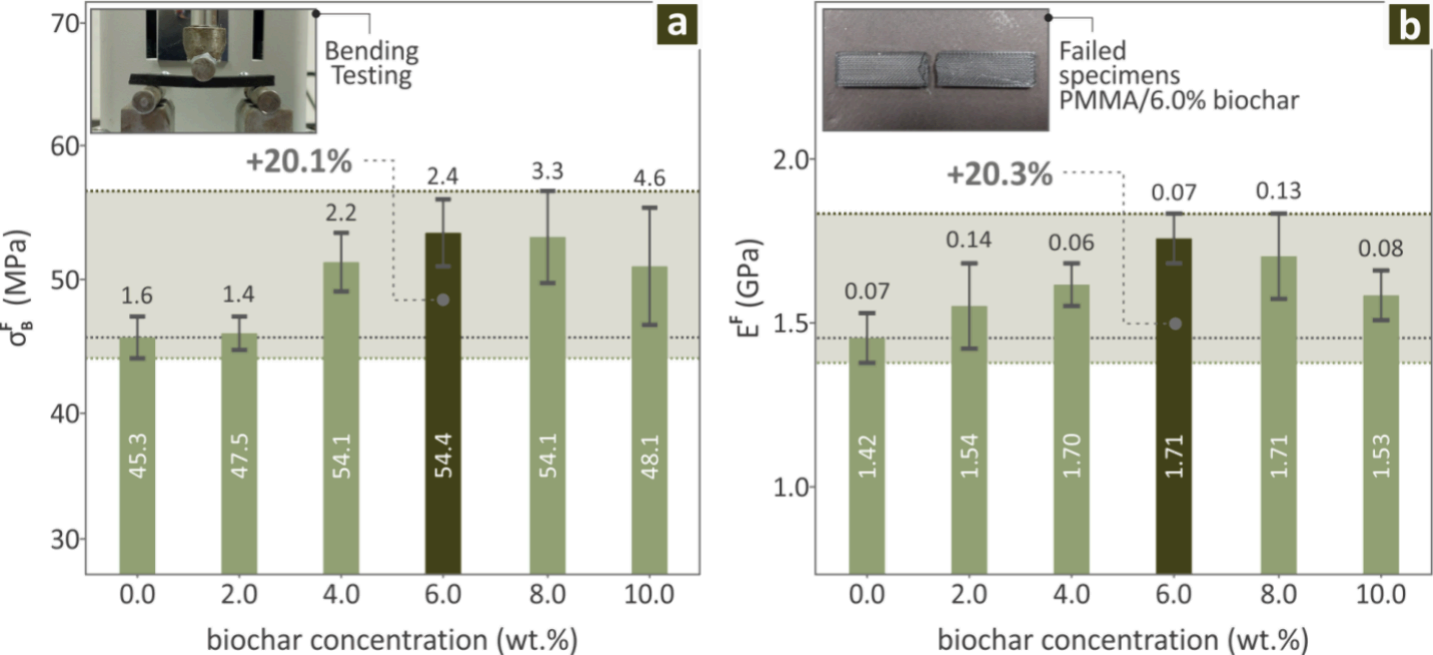
For PMMA/Biochar (0.0–10.0 wt %) bending tested coupons, results with regard to (a) σ_*B*_^*F*^ (b) *E* ^*F*^, as well as images showing the bending testing procedure, and a PMMA/Biochar 6.0 wt % failed specimens, respectively.

The Charpy impact results for the Biochar (0.0–10.0 wt %) corresponding coupons are exhibited in Figure 6a, in bars, indicating the highest levels at PMMA/Biochar 6.0 wt % (17.6% over pure PMMA). The M-H measured levels are shown in Figure 6b, highlighting the Biochar 10.0 wt % sample having the highest levels (15.4% over pure PMMA), while a Vickers pure PMMA imprint is also included.

**Figure 6:**
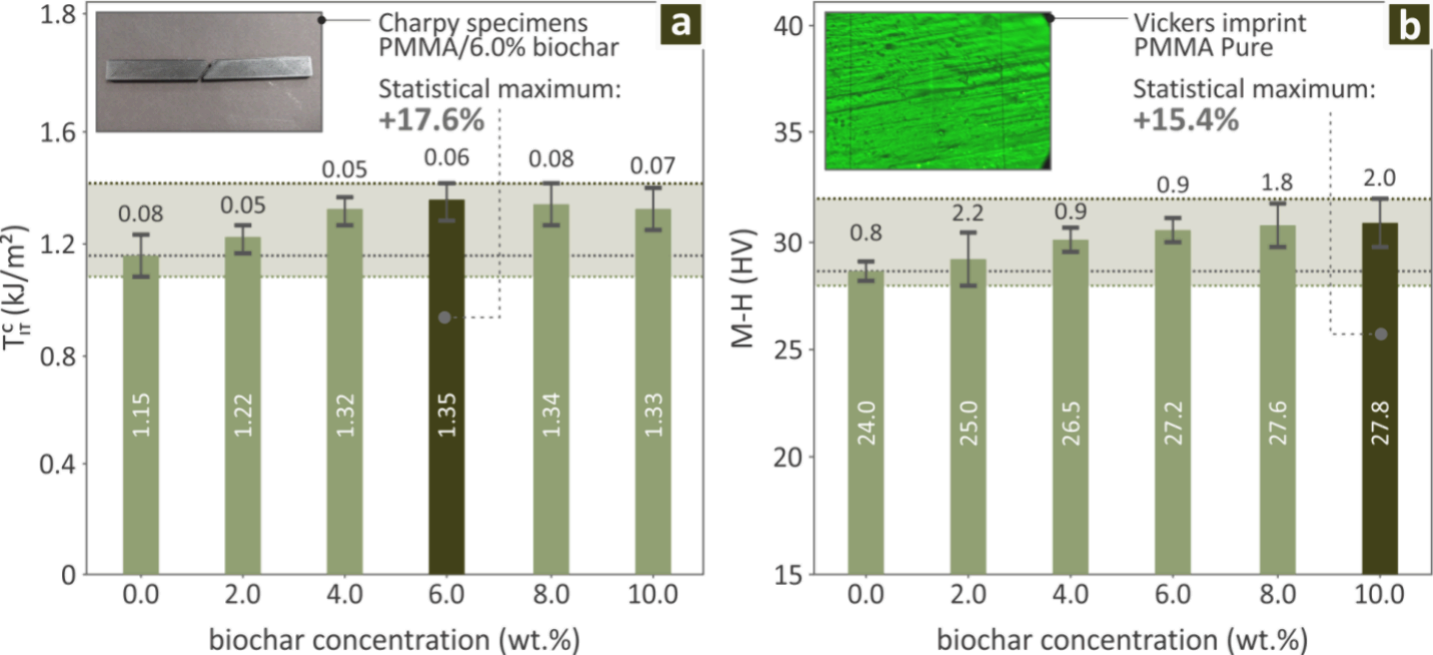
For PMMA/Biochar (0.0–10.0 wt %) tested coupons, results with regard to (a) *T*_*IT*_^*C*^ (b) M-H, as well as images showing a Charpy impact PMMA/Biochar 6.0 wt % failed coupon and a Vickers pure PMMA imprint, respectively

### 3.4 Micro-Computed Tomography Results

Figure 7 shows the structural examination of the PMMA Biochar (0.0–10.0 wt %) samples, showing information for their dimensional deviation and voids. Figure 7a shows the graphs of the dimensional deviation results, which reveal the very good geometrical accuracy of the PMMA/Biochar 6.0 wt % 3D printed samples, where the dimensional deviation seems to be limited in a range closer to zero, than the rest composite samples. Figures 7b and c show the void volume versus void diameter, as well as the pores’ sphericity and compactness, and as a function of the diameter. Fewer voids were observed in PMMA/Biochar 6.0 wt % (647 voids). Figure S4 of the Supporting Information includes additional information on the dimensional deviation and porosity of the created coupons.

**Figure 7:**
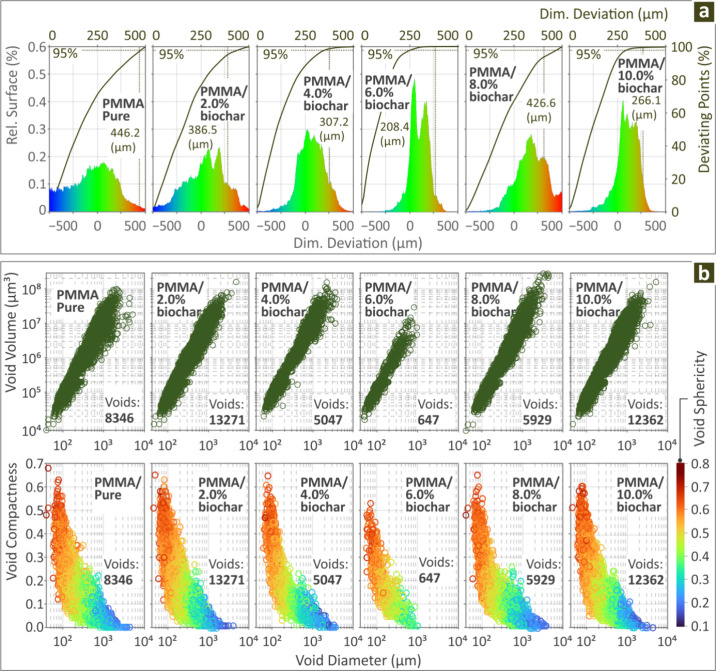
For PMMA/Biochar (0.0–10.0 wt %) samples the structural characteristics in (a) dimensional deviation; (b) void volume vs void diameter and void compactness and sphericity vs diameter graphs.

### 3.5 SEM Morphology

In Figure 8 and Figure 9, the morphological properties of the selected PMMA-biochar compounds are shown at different magnifications, showing their vertical and fractured sections. Figures 8a, d, and g show pure PMMA, PMMA/Biochar 4.0 wt % and PMMA/Biochar 8.0 wt % lateral surfaces (magnified in 150×), revealing a mediocre layering and material distribution, as well as some voids and defects, mostly at PMMA/Biochar 8.0 wt %. Figures 8b, e, and h show the respective fractured regions of the same composites magnified 27×, while Figures 8c, f, and I show them at 5000× magnification. When observing the fracture surfaces from 27× magnification, voids can be detected in the compounds with 4.0 and 8.0 wt % Biochar, while the behavior of the samples is brittle. Figure 9 presents the SEM images of the PMMA/Biochar 6.0 wt % example showing lateral surface pictures in 27× and 150× magnification (Figure 9a and b) as well as fracture surface in 27×, 300×, 1000× and 5000× magnifications (Figure 9c, d, e and f). Remarkably, the fractured surfaces of PMMA/Biochar 6.0 wt % reveal some pores and large voids, while there is a brittle failure response.

**Figure 8:**
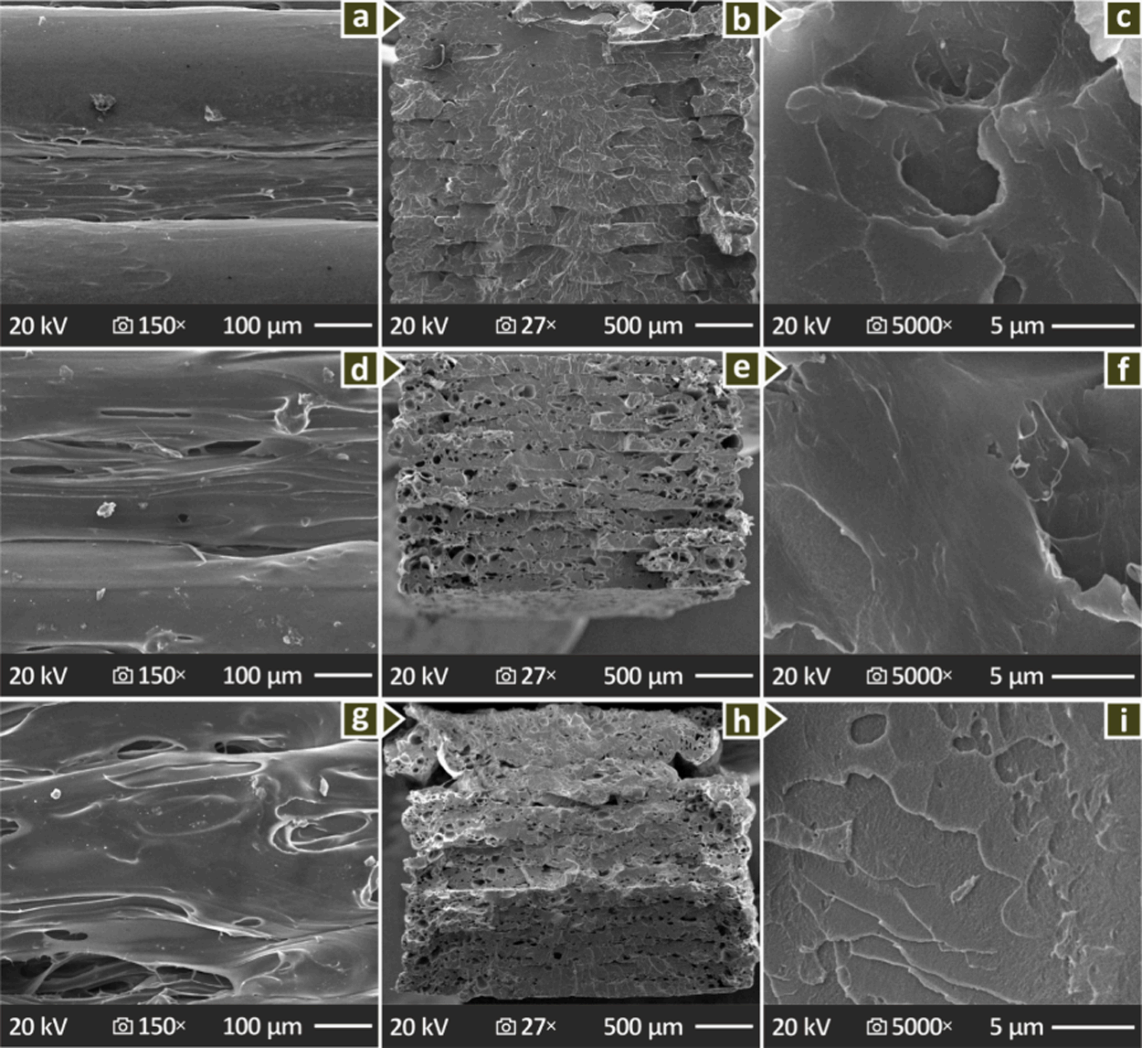
SEM of (a–c) unfilled PMMA, and PMMA/Biochar (d–f) 4.0 wt % and (g–i) 8.0 wt %: lateral (150× magnification) and fractured surfaces (27× and 5000× magnifications).

**Figure 9:**
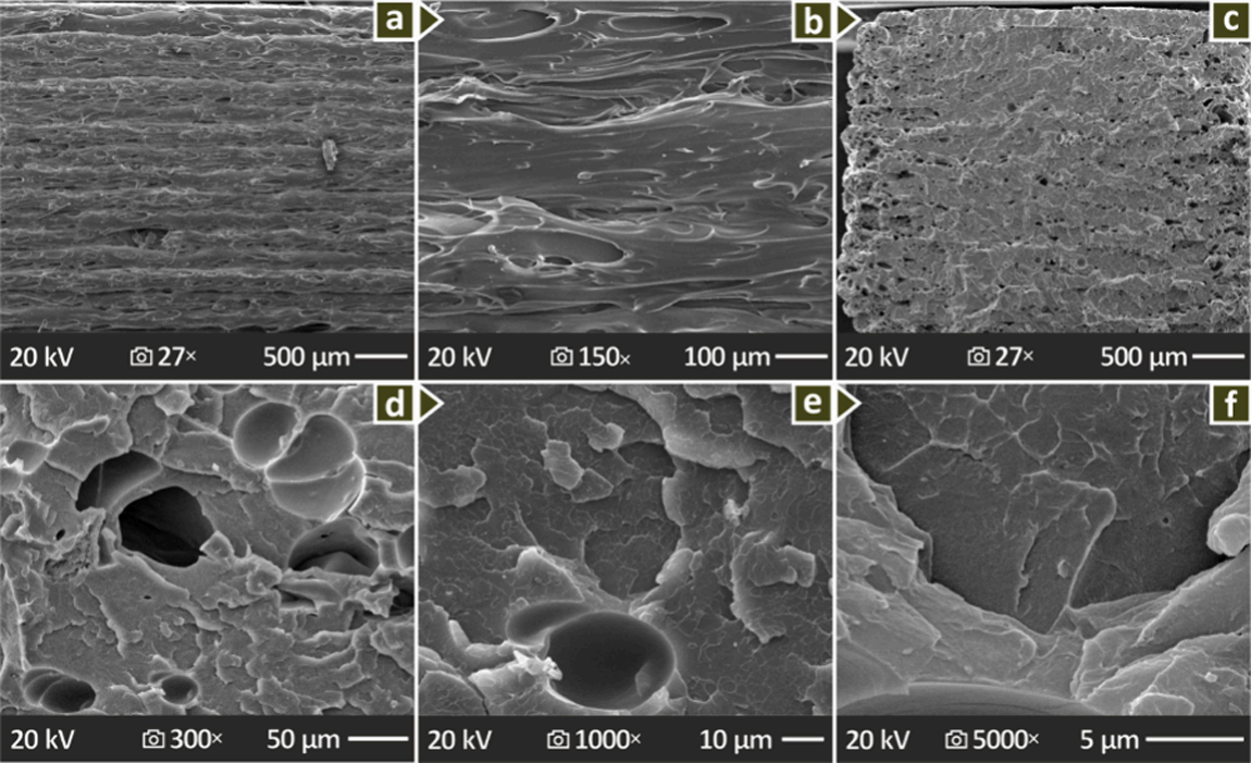
SEM of PMMA/Biochar 6.0 wt % examined coupons, namely, (a, b) lateral section magnified 27× and 150×, and (c–f) fracture region magnified 27×, 300×, 1000×, and 5000×.

## 4 Discussion

An integrated approach to the impact of biochar on the performance of PMMA was developed on the composites created in the raw material, filament, and 3D-printed coupon form. As the filler concentration increased, each mechanical property acted differently, but all the PMMA/Biochar (2.0, 4.0, 6.0, 8.0, and 10.0 wt %) composites had improved performance in relation to pure PMMA. The integration of biochar into PMMA (poly­(methyl methacrylate)) filaments presents several distinct advantages over conventional carbon fillers such as carbon fibers. These advantages include enhanced processing efficiency, improved material performance, reduced environmental impact, and increased cost-effectiveness. From a material processing perspective, biochar’s fine particulate nature facilitates a more uniform distribution compared to the larger, more rigid, and agglomeration-prone carbon fibers. This, thereby enhances the overall mechanical uniformity. Furthermore, biochar is considerably less abrasive than carbon fibers, which significantly benefits the longevity of manufacturing equipment, particularly extruder nozzles used in 3D printing and filament production. In terms of mechanical and thermal properties, although biochar may not achieve the tensile-stress resistance of carbon fibers, it enhances the toughness and thermal stability of the PMMA composite. Its porous, particulate structure provides protection against stress and impact, allowing for more even stress distribution and, under certain conditions, increased filament strength. Notably, being less dense than carbon fibers, biochar contributes to a lighter composite material, which is crucial in applications where weight reduction is a priority. The environmental benefits of utilizing biochar are particularly noteworthy. Produced from biomass through pyrolysis, biochar is a renewable, carbon-negative material, in contrast to carbon fibers, which are manufactured through energy-intensive processes that increase the carbon footprint. The use of biochar supports sustainability goals and promotes a circular economy, especially when sourced from agricultural or forest residues. Economically, biochar is generally much less expensive than carbon fibers, providing manufacturers with a cost-effective alternative while maintaining enhanced material properties. Although carbon fibers offer higher electrical conductivity, biochar can still impart moderate conductivity, suitable for applications such as antistatic coating or basic electromagnetic shielding. Moreover, the properties of biochar can be tailored by selecting specific feedstocks and adjusting pyrolysis parameters, allowing for customized composite formulations to meet application-specific requirements. Overall, biochar represents a more environmentally friendly, cost-effective, and process-efficient alternative to carbon fibers for PMMA composites, provided that maximum strength or conductivity is not the primary requirement. Its inclusion enables more sustainable production without significantly compromising material performance.

In Figure 1 (a), the pure PMMA spectrum exhibits the characteristic peaks of PMMA as are found in the literature. In Figure 1 (b), the Raman spectra of PMMA/Biochar composites across the 0.0 wt % to 10.0 wt % biochar concentration range, with a 2.0 wt % step are shown. With the increasing biochar content, the notable changes observed, are in the intensity and shape of the D-band (∼1350 cm^–1^) and G-band (∼1580 cm^–1^). Those bands are well-known and associated with the biochar’s disordered (D-band) and graphitic carbon structures (G-band). The 6.0 wt % biochar composite presents a specific change in the D/G bands’ intensity ratio compared to other concentrations, suggesting a change in the biochar’s structural order or interaction with the PMMA matrix at this specific composition.

The MFR levels kept decreasing with an increase in the biochar filler percentage, while all of the PMMA composites were between 2.0 and 8.0 wt % were kept around the same value (Figure 2b). Viscosity also decreased by the addition of biochar (in a nonmonotonic way, as it is analyzed below), indicating that no non-Newtonian shear-thinning behavior was found herein (Figure 2a). However, the impact of biochar on PMMA polymer rheology should be considered in relation to 3D printing parameters, which may need adjustment as MFR decreases. The incorporation of biochar as a filler in PMMA matrices for 3D printing applications exhibits a nonmonotonic behavior in melt viscosity with varying filler content. Specifically, at low filler content, viscosity decreases; at intermediate levels, it increases; and at high loadings (8–10 wt %), it decreases again. This behavior is driven by a complex interplay of filler–polymer interactions, dispersion states, and the structural evolution of the composite melt. At low biochar concentrations, the addition of the filler reduces the viscosity of the PMMA matrix. This phenomenon is typically described as a lubrication or plasticizing effect, where biochar particles act as spacers for the polymer chains, reducing interchain entanglements and enhancing chain mobility. In this scenario, the dispersed particles increase free volume and reduce the melt’s frictional resistance. Similar performance has been observed in other filler systems, where low concentrations of carbonaceous additives reduce melt viscosity by interfering with polymer entanglements. Increasing the biochar concentration to an intermediate level results in a rise in composite viscosity. This effect is usually linked to the onset of particle–particle interactions and the formation of a transient network of fillers within the polymer matrix. These interactions constrain polymer chain mobility by creating physical barriers and increasing the system’s hydrodynamic volume. The resulting microstructural network resists shear deformation, thereby elevating the apparent viscosity. Such behavior has been noted in carbon black-filled polyamide composites, where intermediate filler concentrations favored the formation of percolated structures that restricted flow. However, at higher biochar concentrations—particularly 8 and 10 wt %—viscosity decreases again. This seemingly counterintuitive decrease can be attributed to the agglomeration of biochar particles, which reduces their effective surface area and interaction with polymer chains. High filler loading can also lead to the breakdown of existing network structures, weakening the composite melt’s viscoelastic response. Under shear conditions typical in fused filament fabrication (FFF) or other extrusion processes, biochar agglomerates are expected to align with the flow direction, facilitating slippage and reducing resistance. Similar tendencies have been observed in polymer composites used for additive manufacturing, where elevated filler loadings caused network collapse and increased flow due to poor dispersion and shear orientation. Overall, the viscosity response of PMMA–biochar composites at various filler loadings transitions from a lubrication-dominated regime at low concentrations, through a structure-forming regime at intermediate concentrations, to network disruption and alignment at high loadings. Understanding this rheological response is valuable for optimizing printability and mechanical performance in biochar-reinforced polymer composites.^113–117^

The introduction of biochar in the PMMA reduces the MFR, but the values are rather stable for concentrations up to 8 wt %. As the concentration of biochar increases, particles may initiate the formation of filler networks within the PMMA matrix. These networks can establish physical barriers that entrap the polymer chains, thereby impeding the flow of the melt. The network structure introduces additional resistance to shear flow, consequently contributing to the decreased melt flow rate. As MFR decreases indicates an increase in the viscosity, this seems to contradict the viscosity measurements. Still, such differences are expected due to the different nature of the two rheological measurements (viscosity vs shear rate and MFR). Viscosity versus shear rate assessments and melt flow rate evaluations are both utilized to examine the flow characteristics of materials, particularly polymers, yet they serve distinct purposes and are conducted through different methodologies. Viscosity versus shear rate assessments provide an accurate representation of a material’s flow behavior, especially for non-Newtonian fluids such as molten plastics, whose viscosity can be shear rate-dependent. The results are crucial for understanding how the material behaves under different processing conditions, such as during extrusion or injection molding, where shear rates can vary significantly. Conversely, the MFR is a more straightforward test providing a single reading loosely correlated to the molecular weight of the material—increasing MFR readings generally suggest decreasing molecular weight polymers. This does not apply herein as a compound is tested not a single polymer. MFR is widely employed in quality control and for comparing different batches of the same material, thus it was implemented herein. At the highest concentration of 10 wt % MFR is reduced.^113,114^ At higher concentrations, particle agglomeration leads to a decrease in both viscosity and MFR.^118–121^

The FR and IDT (Figure 3d) levels were affected by the incorporation of Biochar to PMMA. The FR constantly increased, as expected, due to increased biochar content in the compounds. The introduction of biochar decreased the IDT; still, the decrease was not high enough to assume that the biochar filler could compromise the thermal stability of the PMMA polymer. TGA was carried out as it is an essential technique for the characterization of polymeric composites utilized in 3D printing. Its importance is derived from its capacity to provide comprehensive insights into the thermal stability and decomposition patterns of new compounds, which have a direct impact on their performance during the printing process. The effect of biochar on the thermal stability of PMMA was evaluated through these measurements. Furthermore, given that MEX 3D printing necessitates the heating of materials to specific temperatures for extrusion, it is imperative to ascertain the temperature at which these materials commence thermal degradation. TGA facilitates the identification of the degradation temperature, thereby establishing a safe thermal processing window to ensure the compounds’ integrity during printing. Additionally, TGA assists in the analysis of the composition of polymeric materials, such as the quantification of additives through the residual mass. There are also other important aspects in which TGA findings can be useful but mainly apply to unfilled polymers, not compounds. The TGA findings presented in Figure 3 show that the FR increases with the increase of the filler content in the compounds, which is the expected outcome, verifying the presence of increased biochar in the compounds. Furthermore, it was verified that the extrusion temperatures (filament production and parts 3D printing) do not cause any degradation in the compounds. Finally, it was found that the introduction of biochar does not compromise the thermal stability of the PMMA thermoplastic. The IDT of the PMMA was reduced by the addition of the biochar. The reduction is negligible for the 2, 4, and 8 wt % compounds (less than 3 °C). The highest IDT reduction of about 10 °C was found for the 6 wt % compound, which also cannot be considered a significant one.

Tg initially increased up to 6.0 wt % biochar content in the compounds and then started to decrease (Figure 3). When filler content is low, robust interactions (such as hydrogen bonds and van der Waals forces) between polymer chains and filler particles can limit polymer chain movement, causing an elevation in tg. Well-distributed filler particles can fill free spaces within the polymer matrix, restricting segmental motion and consequently increasing tg. As filler loading increases, particles tend to form clusters, resulting in phase separation and weaker polymer–filler interactions. This effect diminishes the rigid interfacial influence and lowers tg. High filler content may disrupt polymer chain entanglements, reducing overall rigidity and consequently lowering tg.^122,123^

Glass transition temperature (Tg) is a critical thermal property of polymeric materials that substantially affects their performance in 3D printing applications. Tg denotes the temperature range at which a polymer transitions from a rigid, glassy state to a pliable, rubbery state. In MEX, Tg defines the process window and influences layer adhesion, dimensional stability, and mechanical properties.^124^ For optimal printing, the material must exceed its Tg to ensure adequate flow and interlayer bonding. Conversely, if the Tg is insufficiently high relative to the operating environment, printed components may experience deformation, creep, or mechanical failure under stress.^125^ Polymers with elevated Tg values are preferred in high-performance applications due to their superior thermal and dimensional stability.^126^ Consequently, selecting polymeric materials based on their glass transition temperatures is essential for optimizing the printability, mechanical reliability, and long-term performance of 3D objects. Herein, the 3D printing process was performed at temperatures higher than the measured Tg for all compounds. The introduction of biochar in the PMMA thermoplastic increased the Tg of the compounds for all filler concentrations. The 2 and 4 wt % composites exhibited an increase of about 8 °C in the Tg (from 106.6 to 114.5 °C and 114.2 °C respectively). In the higher loaded compounds of 8 and 10 wt % rather similar Tg values were found. They were about 2 °C lower than the 2 and 4 wt % composites (112.4 and 112.2 °C respectively. The highest increase in the Tg exhibited in the 6 wt % compound with about 10 °C higher temperature than the pure PMMA (116.2 °C). An increase in the Tg due to the introduction of additives in polymeric matrices is expected, as reported in the literature. An increase up to 30 °C can be considered moderate, while increases higher than 30 °C are characterized as significant.^127^

SEM images (Figure 8, Figure 9) showed that the introduction of biochar worsens the uniformity of the layer structure when observing the samples from the side. Still, this has no negative effect on the mechanical properties, as the respective experimental procedure showed. Differences in the lateral SEM images can be attributed to different rheology in the compounds, which affect the 3D printing structure, in terms of its quality. This can have an effect on the layer formation, the presence of defects, the uniformity of the layer thickness, and the layer fusion, among others. The difference in the lateral surfaces of the 4, 6, and 8 wt % compounds is not that high. Probably it was the zoom region selected that led to this comment. Any differences can be attributed to the differences in the viscosity between the different biochar content compounds. All compounds were 3D printed with the same settings for comparison purposes, therefore, the difference in the flow behavior between the compounds is expected to affect the formation of the 3D printing structure. This is evident, as the pure PMMA shows a uniform layer formation, with a smooth external surface, very good layer fusion, and no defects or variations in the structure. The compounds, on the other hand, show a surface pattern that is not smooth, with layer formation and uniformity of the thickness variating between the compounds. By inspecting the fraction region of the samples, in all cases, a brittle fracture mechanism can be observed.

When examining the structural behavior of the PMMA samples, the dimensional deviation results indicated PMMA/Biochar 6.0 wt % as the most accurate one geometrically wise, while the PMMA/Biochar 10.0 wt % also presented good results in this metric (geometrical deviation). With regard to the voids measured, PMMA/Biochar 6.0 wt % composite revealed a lower number of voids (647), while PMMA/Biochar 2.0 wt % was characterized by a large amount of voids (13271). The PMMA/Biochar 6.0 wt % compound was the one with the most improved mechanical performance among the concentrations tested. Therefore, a correlation between porosity and mechanical performance can be safely assumed, which is the expected response when consulting the respective literature.^128–130^ Still, the superior properties of the 6 wt % composite are not attributed only to the fewer voids in the 3D printing structure. Other aspects contributed to this outcome, such as the formation of a filler network in the composite, that reduces polymeric chains’ mobility, the interaction between the filler and the matrix, and the rheology of the compounds among others.

Regarding the mechanical tests, σ_*B*_^*T*^ increased up to a point (6.0 wt % concentration, Figure 4) and then began to decrease again, which also happened in the case of *T* ^*T*^, σ_*B*_^*F*^, *E* ^*F*^ and *T*_*IC*_^*C*^. For the rest of the tested mechanical properties, namely *E* ^*T*^ and M-H (Figure 6), the distinguished concentrations were namely 8.0 and 10.0 wt %. The improvement of all the tensile and bending (Figure 5) properties was over 20.0% compared to the performance of pure PMMA. Overall, the 6.0 wt % concentration can be concluded to be the optimum one in this research. The Supporting Information of this work contains Figure S5, which summarizes the mechanical properties investigated in the four spider graphs and highlights the maximum values as well as the respective composites at which they were detected. Overall, the introduction of biochar did not alter the behavior of the samples. This can be derived from the fact that the samples with the highest strength also had the highest toughness in the respective experiments. The enhanced mechanical properties observed for the 6.0 wt % biochar composite, specifically the >20% increase in tensile and flexural strength, correlates also with the Raman data. The changes in the D/G band intensity ratio at 6.0 wt % biochar suggest an optimal balance between the biochar’s structural order and its interaction with the PMMA matrix, facilitating effective stress transfer and reinforcement.

For the determination of these mechanical properties, the instructions of the respective standards were applied. The mechanical test standards instruct the testing of five samples per case, the ignorance of possible extreme values in a sample, and the calculation of the mean value out of the tested samples as the value of the respective mechanical property. This was applied herein. The calculated values presented should be considered the respective experimental findings according to the instructions. Therefore, apart from their values, they show the reinforcement pattern with regard to the biochar content in the compounds for each property. The deviation is presented to show the differences between the samples of the same batch. Significant variability in the mechanical testing of 3D-printed polymers is both common and anticipated due to the inherent characteristics of additive manufacturing processes. Various aspects affect this response, such as the anisotropic nature of 3D-printed components. The deposition of material in layers results in weaker interlayer bonding compared to intralayer bonding, leading to mechanical properties that are direction-dependent.^125^ In addition to anisotropy, inconsistencies in material deposition processes further contribute to mechanical variation. Parameters such as nozzle temperature, feed rate, and printer calibration can introduce defects like voids, under-extrusion, or uneven layer adhesion.^131^ Printer settings such as layer thickness, infill density, and print speed further contribute to variability. It was observed that minor adjustments in these settings can lead to significant changes in strength and failure behavior.^132^ Additionally, the quality and consistency of the filament itself can vary between manufacturers or even from spool to spool.^133^ Collectively, these factors result in mechanical test data on 3D-printed polymers typically exhibiting a higher standard deviation than conventionally manufactured plastics. Coefficients of variation for tensile strengths can range from 5–20% for well-controlled, high-quality prints, but may exceed 30% with poor process control or lower-grade equipment. The results provided are well within the limits of good-quality commercial filaments.

Another aspect that should be noted is that PMMA is a clear (transparent) material commonly used in optics and related applications. When 3D printed, it becomes blurred, due to the 3D printing structure, still, it remains transparent, as shown in the images of the filament in the supplementary file. The addition of biochar turns the material black, due to the presence of carbon. Therefore, the mechanical strength was improved, but the material loses this feature and cannot be further used for such types of applications requiring transparency from the material. This is one of the limitations of the compounds introduced. Still, there are numerous other areas of application in which PMMA can be used. These include catalysis, sensors, solar cells, photodetectors, and various industrial applications, as already stated. In the medical field, the presence of carbon again requires additional tests, for cytotoxicity^134,135^ for example among others to confront the respective standards of each application.

To the best of the authors’ knowledge, this study is the first to produce and characterize PMMA/biochar filaments and their 3D-printed specimens in this manner. As mentioned, both are environmentally friendly materials, as PMMA was derived from waste and biochar from olive tree prunings. While the scientific community has explored other systems for 3D-printing applications as the introduction of this paper noted. These studies utilized the same biochar grade and similar fabrication techniques as the current research, without additional additives.

Table 2 presents a comparison of mechanical properties from existing literature, highlighting the reinforcement effect of the specific biochar grade on different polymers, including the current findings on PMMA. The results for PMMA are comparable to most of the matrices tested so far, while the PMMA herein also has an eco-friendly character since it was recycled from waste. The reinforcing effect was slightly higher on HDPE^100^ and slightly lower on PETG.^99^ Interestingly enough, the optimal biochar loading was similar in all cases. Increasing the biochar content in PMMA beyond the optimal point led to processability issues, making sample preparation more challenging.

**Table 2:** Evaluating the Effectiveness of Biochar as a Strengthening Component in Material Extrusion 3D Additive Manufacturing for Widely Used Polymers and Recycled PMMA

increase (%)	PMMA	PLA.^94^	PETG^99^	PP^97^	HDPE^100^	ABS^101^
Young modulus	20.5	25.8	14.8	24.3	29.5	25.5
tensile strength	21.9	20.9	17.8	28.4	37.8	24.9
flexural strength	20.1	14.1	15.9	19.7	35.9	21.0
opt. loading (wt %)	6.0	4.0	6.0	4.0	6.0	4.0

## 5 Conclusions

This research aimed to introduce environmentally friendly composites for the MEX AM method with enhanced mechanical performance. To achieve this objective, a nature-sourced filler, specifically biochar, was utilized for reinforcing a recycled PMMA thermoplastic derived from waste trimmings. Valuable information was obtained from the execution of this investigation regarding the capabilities of PMMA/biochar composite materials under different biochar concentrations (0.0–10.0 wt %). The fabrication of the raw composite materials into filaments and subsequently into the respective coupons was intended to create samples suitable for mechanical, rheological, and thermal investigations, as well as structural and morphological characterization. The addition of biochar to PMMA provided a composite with improved properties at the majority of the filler concentrations. The key findings can be summarized as follows:

- PMMA/Biochar 6.0 wt % was the composite which demonstrated an increase in most of the properties compared to pure PMMA namely σ_B_^T^ (21.9%), T^T^ (20.2%), σ_B_^F^ (20.1%), E^F^(20.3%) and T_IT_^C^ (17.6%).
- PMMA/Biochar 8.0 wt % exhibited significant E^T^ performance improvement (20.5%) and PMMA/Biochar 10.0 wt % displayed the highest M-H levels (15.4%).
- Quality metrics of the 3D printing structure (geometrical accuracy and porosity) were improved by the addition of biochar in the PMMA matrix. PMMA/Biochar 6.0 wt % was the composite with the most improved quality indicators, showing an effect of the parts’ quality on the mechanical performance. Still, aspects, such as the filler/matrix interactions among others, led to this outcome.
- The rheology of PMMA was also affected as expected, with a decrease in the viscosity of the compounds.
- The thermal stability was not notably affected by the introduction of biochar to the PMMA matrix.
- The SEM images predominantly revealed brittle character, the layering was not entirely well-distributed, and porosity and voids were detected.

The results were promising for the future utilization of these environmentally friendly composites in 3D printing and their prospects. Most of the evaluated metrics were improved by the addition of biochar in the PMMA thermoplastic. Further research could be conducted to investigate additional properties or attempt to optimize the 3D printing parameters, due to the change in rheology.

## Supplementary Material



## Data Availability

The raw/processed data required to reproduce these findings cannot be shared because of technical or time limitations.

## Notes

(Figure S1) (a) PMMA material preparation by PMMA sheet trimming shredding, (b, c) raw materials preparation and dehydration, (d, e, f, g) extrusion, dehydration, diameter monitoring, inspection, and mechanical testing of filaments, (h) coupon 3D printing by AM, (i, j) mechanical testing of the coupons, (k) elemental, rheological and thermal testing, as well as (l) morphology examination through SEM; (Figure S2) biochar material SEM pictures magnified in (a) 1000×, (b) at 80,000, and (c) at 130,000× magnification; (Figure S3) (a) pure PMMA filament inspection and diameter monitoring, (b) tensile strength levels of all PMMA/Biochar filaments, (c) PMMA/Biochar 6.0 wt % filament inspection and diameter monitoring, (d) tensile modulus of elasticity levels of all PMMA/Biochar filaments; (Figure S4) stress-to-strain curves of (a) filament tensile, (b) specimen tensile, (c) specimen flexural behavior; (Figure S5) utilized 3D printing parameters, illustrations of the models designed for the various specimens and their dimensions, as well as images from the fabricated tensile, bending, and Charpy Notched specimens; (Table S1) PMMA pure Raman peaks (significant) and the respective assignments; (Figure S6) structural characteristics of PMMA/Biochar 3D printed samples, namely, (a, b) dimensional deviation of a PMMA/Biochar 6.0 wt % specimen by color-coding mapping, (c) dimensional deviation levels in bars of all PMMA/Biochar composite samples, (d, e) representation of PMMA/Biochar 4.0 wt % volume through color-coding mapping, (f) bars of the porosity levels for all of the PMMA/Biochar composite samples; (Figure S7) spider-shaped graphs presenting a summarization of (a) tensile strength, (b) tensile modulus of elasticity, (c) flexural strength, and (d) flexural modulus of elasticity, for all of the PMMA/Biochar composite samples (PDF)
