# Supplementary material for: Cyclic Economy-Driven Composites for Material Extrusion Three-Dimensional Printing: Poly(methyl methacrylate) from Recycled Scrap with Optimized Biomass-Derived Biochar Filler Content
Source: ACS Omega. 2025 Jun 6;10(23):24980–95. doi: 10.1021/acsomega.5c02525 (PMC12177765; doi:10.1021/acsomega.5c02525)
Supplement: Supplementary file 1 [file ao5c02525_si_001.pdf]

Supplementary for article

Cyclic economy-driven composites for MEX 3D printing: PMMA from recycled scrap with optimized biomass-derived biochar filler content

Nectarios Vidakis [a], Nikolaos Michailidis [b, c], Dimitrios Kalderis [d], Emmanuel Maravelakis [d], Vassilis Papadakis [e, f], Apostolos Argyros [b, c], Nikolaos Mountakis [a], Maria Spyridaki [a], Nektarios Nasikas [g], Markos Petousis [a*]

[a] Department of Mechanical Engineering, Hellenic Mediterranean University, Heraklion 71410, Greece

[b] Physical Metallurgy Laboratory, Mechanical Engineering Department, School of Engineering, Aristotle University of Thessaloniki, 54124 Thessaloniki, Greece

[c] Centre for Research & Development of Advanced Materials (CERDAM), Center for Interdisciplinary Research and Innovation, Balkan Centre, Building B', 10th km Thessaloniki-Thermi road, 57001, Thessaloniki, Greece

[d] Department of Electronic Engineering, Hellenic Mediterranean University, Chania 73133, Greece

[e] Department of Industrial Design and Production Engineering, University of West Attica, 122 43 Athens, Greece

[f] Institute of Electronic Structure and Laser, Foundation for Research and Technology–Hellas, N. Plastira 100m 70013 Heraklion, Greece

[g] Division of Mathematics and Engineering Sciences, Department of Military Sciences, Hellenic Army Academy, 16673 Vari, Attica, Greece

* Corresponding author, E-mail: markospetousis@hmu.gr (Markos Petousis), Tel.: +302810379227

Abstract

Environmentally friendly materials are emerging materials that find applications in an increasing number of cases when being three-dimensional printed (3D-P), as they can provide many possibilities and offer unique properties to fulfill industrial needs and requirements. As part of that effort, recycled (from sheet trimmings waste) poly (Methyl Methacrylate) (PMMA) and (nature-sourced) biochar were selected to be combined and examined herein. Composites with Biochar concentration in the 0.0 wt. %-10.0 wt. % (2.0 wt.% step) range, were assessed. Compounds were extruded into filaments, which fabricated coupons (material extrusion 3D-P) for the tests that followed. The samples were experimentally evaluated for their characteristics related to mechanical, chemical, rheological, and thermal behavior, as well as for their structure and morphology. Mechanical testing included tensile, bending, and Charpy Notched coupons' investigation. The microhardness was also measured. In addition, quality characteristics were assessed through porosity and dimensional deviation data analysis. The composite distinguished in relation to pure PMMA was PMMA/Biochar 6.0 wt. % (>20% increase in the strength on the tensile and flexural experiment), as most of the investigated properties revealed their greatest values in that Biochar concentration. Furthermore, the eco-friendly

biochar addition positively affected most of the recycled PMMA characterization metrics assessed, showing potential for the environmentally friendly composites developed herein for the 3D-P process.

S.1. Methodology

A brief research work presentation is exhibited in Figure S1, including the treatment of PMMA and Biochar, the filaments extruded from them, the coupons fabricated from the filaments, and their tests. Figure S1a refers to the preparation of PMMA trimmings through shredding, while Figure S1 (b-c) shows the weighting and dehydration of the two materials, Figure S1 (d-g) the extrusion, dehydration, and mechanical testing of the produced filaments; Figure S1h illustrates the fabrication of the coupons and Figure S1 (i-l) the testing of the created samples (mechanical, elemental, rheological, thermal, and morphological).

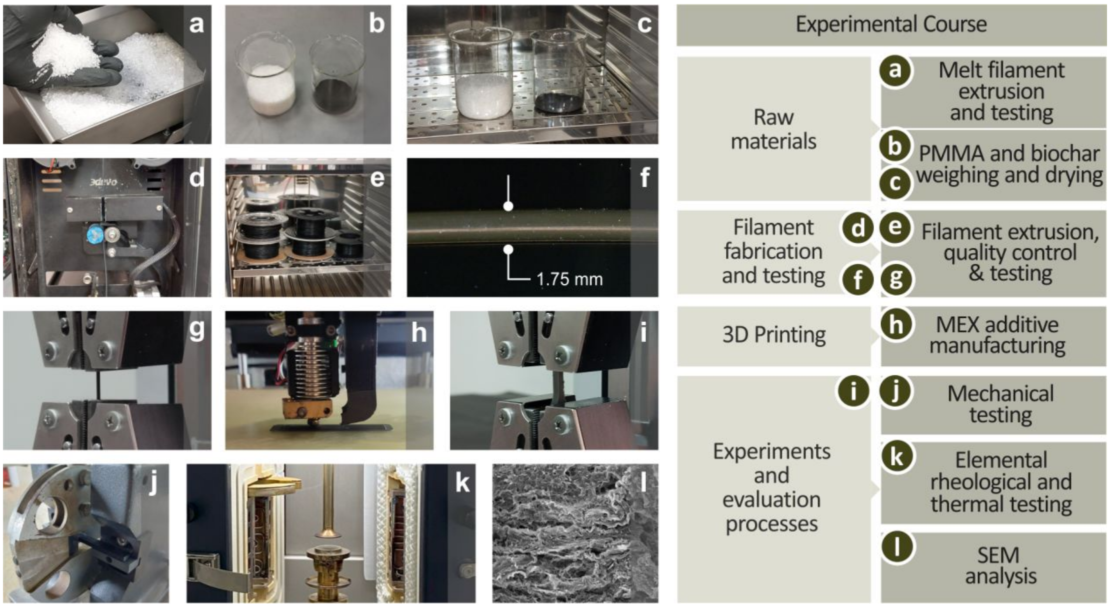

Figure S1. (a) PMMA material preparation by PMMA sheet trimming shredding, (b, c) raw materials preparation and dehydration, (d, e, f, g) extrusion, dehydration, diameter monitoring, inspection, and mechanical testing of filaments, (h) coupon 3D printing by AM, (i, j) mechanical testing of the coupons, (k) elemental, rheological and thermal testing, as well as (l) morphology examination through SEM

S.2. Biochar particles morphology

Figure S2 shows SEM illustrations of the biochar material (particles) at 1000×, 80,000×, and 130,000× magnifications (images taken with a model named JSM-IT700HR, which is a field emission SEM, from the Jeol Ltd. company, located in Tokyo, Japan, gold-sputtered particles, 20kV, high-vacuum mode).

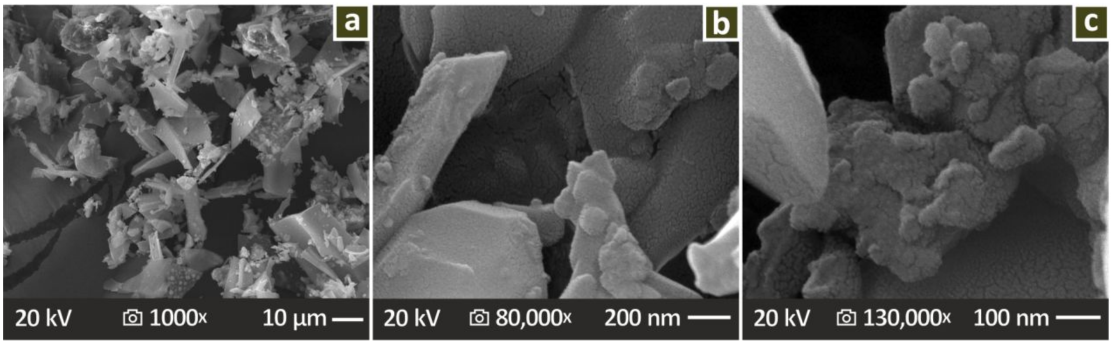

Figure S2. Biochar material SEM pictures magnified in (a) 1000×, (b) at 80,000, and (c) at 130,000× magnification.

S.3. Filament testing and quality assessment

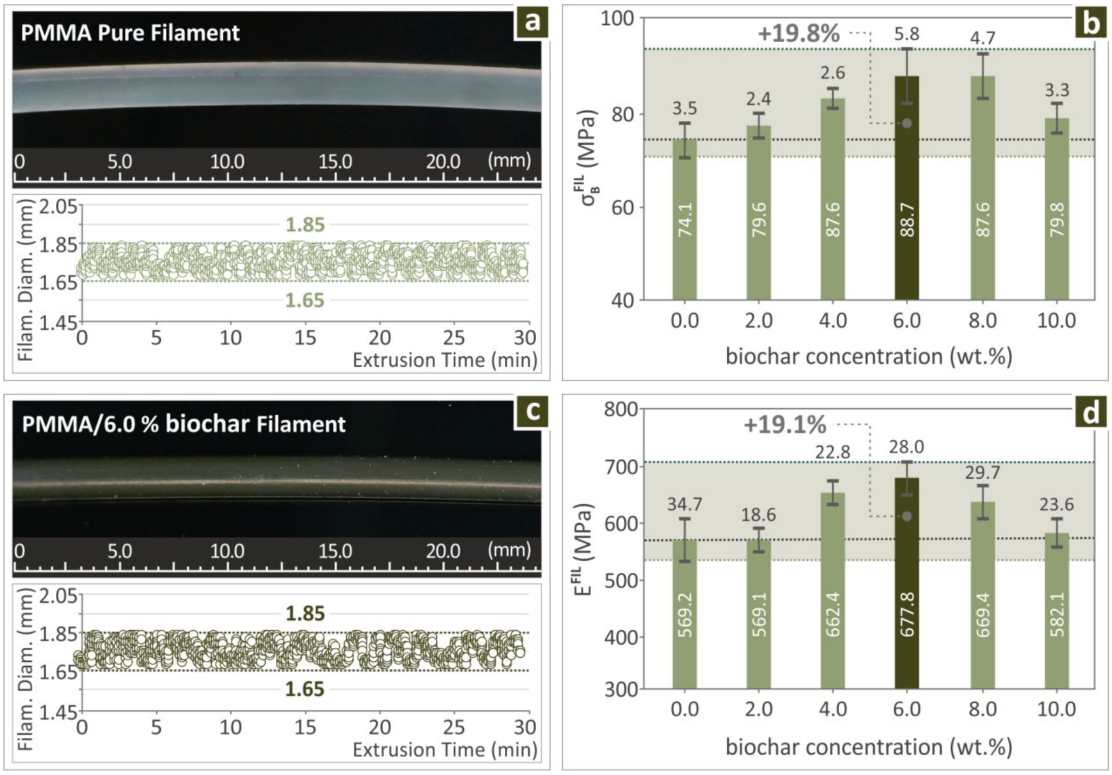

Figure S3. (a) Pure PMMA filament inspection and diameter monitoring, (b) tensile strength levels of all PMMA/ Biochar filaments, (c) PMMA/ Biochar 6.0 wt. % filament inspection and diameter monitoring, (b) tensile modulus of elasticity levels of all PMMA/ Biochar filaments

S.4. Stress vs. strain graphs

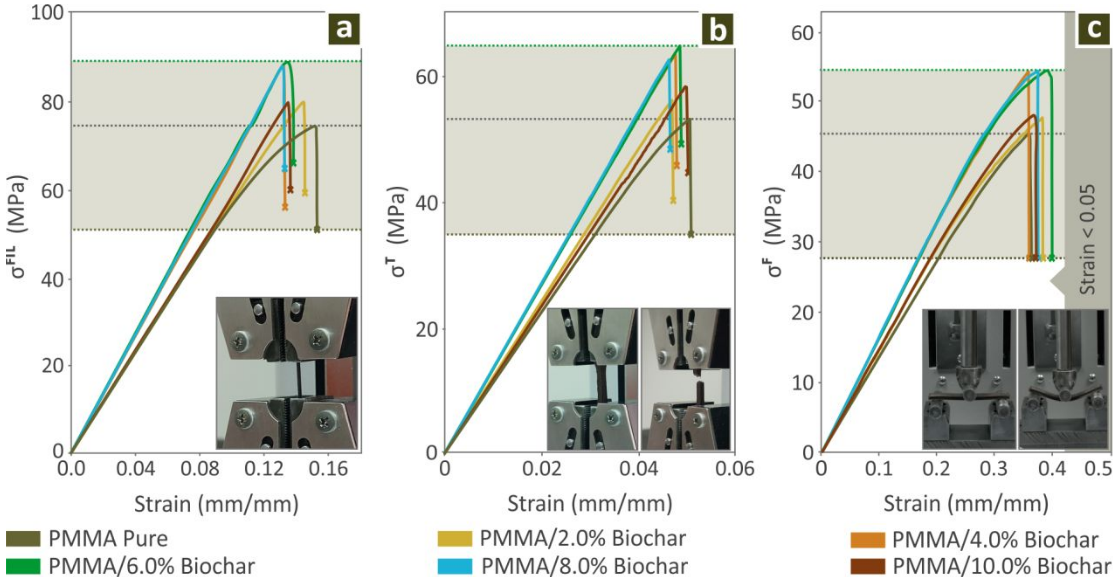

Figure S4. Stress to strain curves of (a) filament tensile, (b) specimen tensile, (c) specimen flexural behavior

S.5. 3D printing settings and samples' geometry

Parameters	Value	Units
PMMA biochar content	0.0–2.0–4.0–6.0–8.0–10.0	%
Printing orientation	±45	deg
Nozzle temperature	200	℃
Bed temperature	40	℃
Layer thickness	0.2	mm
Number of perimeters	2	–
Fill density	100	%
Travel speed	40	mm/sec

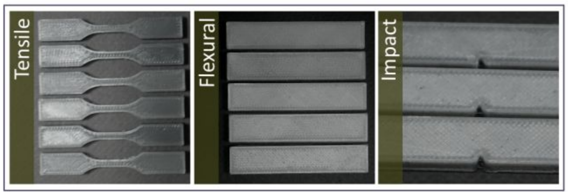

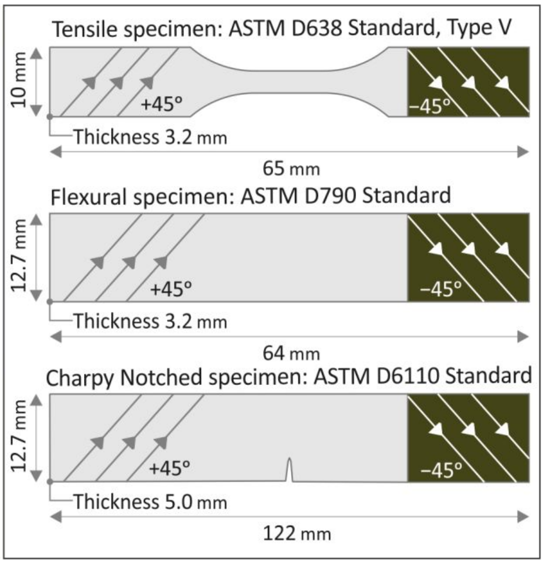

Figure S5. The utilized 3D printing parameters, illustrations of the models designed for the various specimens and their dimensions, as well as images from the fabricated tensile, bending, and Charpy Notched specimens

S.6. Raman spectroscopy, rheology, and thermal properties

A LabRAM HR Raman Spectrometer (HORIBA Scientific, Kyoto, Japan) was used to acquire Raman spectra. A 532 nm solid-state laser module was selected for excitation with a maximum output power of 90 mW. The Raman spectral resolution was approximately 2 cm^{-1} achieved using a grating with 600 grooves. An Olympus objective lens (LMPlanFL N) with a numerical aperture of 0.5 delivered light onto the sample while also collecting the Raman signals. The 50X magnification objective lens operated at a 10.6 mm working distance. A Neutral Density filter with 3.2% transmittance limited the

laser power, which was measured to be 1.2 mW for the sample. The measurement volume was measured to be 1.7 µm laterally and 2 µm axially. The Raman spectra collected were between 50 and 3900 cm^{-1}, which was achieved using three optical windows. Each measurement point had an exposure time of 10 s, with five accumulations. The irradiated areas were then visually inspected to ensure that no discoloration or degradation was observed owing to laser irradiation.

Raw Raman data were processed using LabSpec software (HORIBA, Kyoto, Japan). Each acquired spectrum was processed using the same methodology: a) Cosmic rays were removed; b) signal denoise with 5 points kernel; c) background removal using an 8th-grade polynomial; d) spectra were recalibrated by the 810 cm^{-1} peak, and e) spectra were normalized by the maximum peak.

The related Raman peaks from the unfilled PMMA are depicted in Table S1. Values were extracted from the respective bibliography together with their reference.

Table S1. PMMA pure Raman peaks (significant) and the respective assignments

Wavenumber (cm^{-1})	Intensity	Raman peak assignment
598	Strong	C-COO vibration, C-C-O symmetric vibration [1]
734	Small	C-H out-of-plane bending [2,3]
811	Strong	CH_2 vibration [1]
844	Small	Phenyl ring vibration [4]
911	Small	C-H in-plane bending [3]
964	Strong	O-CH_3 rocking [1]
985	Strong	C-C and C-O vibration [5]
1120	Medium	Skeletal vibrations, C–C bonds [1,5]
1181	Small	Skeletal vibrations, C–O– C, C-COO bonds [1,2,5]
1240	Small	C-O-C stretching [3]
1326	Small	C-O-C stretching [3]
1449	Strong	C-H_3 deformation [1,3]; C-H_2 deformation [2,3]; C-H_3 symmetric bending [3,4,6];
1727	Strong	C = O bond [1,7] C-O-C symmetric stretching [8]
2843	Medium	O-CH_3 vibration [1]
2951	Strong	CH_2 and C-H asymmetric stretching [1,5]
3000	Medium	C-H stretching [3]

The rheological investigation was based on viscosity and MFR tests (ASTM D1238-13), and the data were extracted from the rheometer results from TA instruments named DHR-20 Discovery Hybrid Rotational Rheometer (New Castle, DE, USA). The device had a parallel-plate configuration (25 mm diameter) and an environmental test chamber (temperature-controlled). Thermal inspection was performed using TGA (temperature range of 20-550 °C) and DSC (30 and 270 °C) using a Perkin

Elmer Diamond TGA/DTGA from Perkin Elmer (Waltham, U.S.) and a DSC 25 from TA Instruments (New Castle, U.S.), respectively.

S.7. Micro-computed tomography: dimensional deviation and porosity

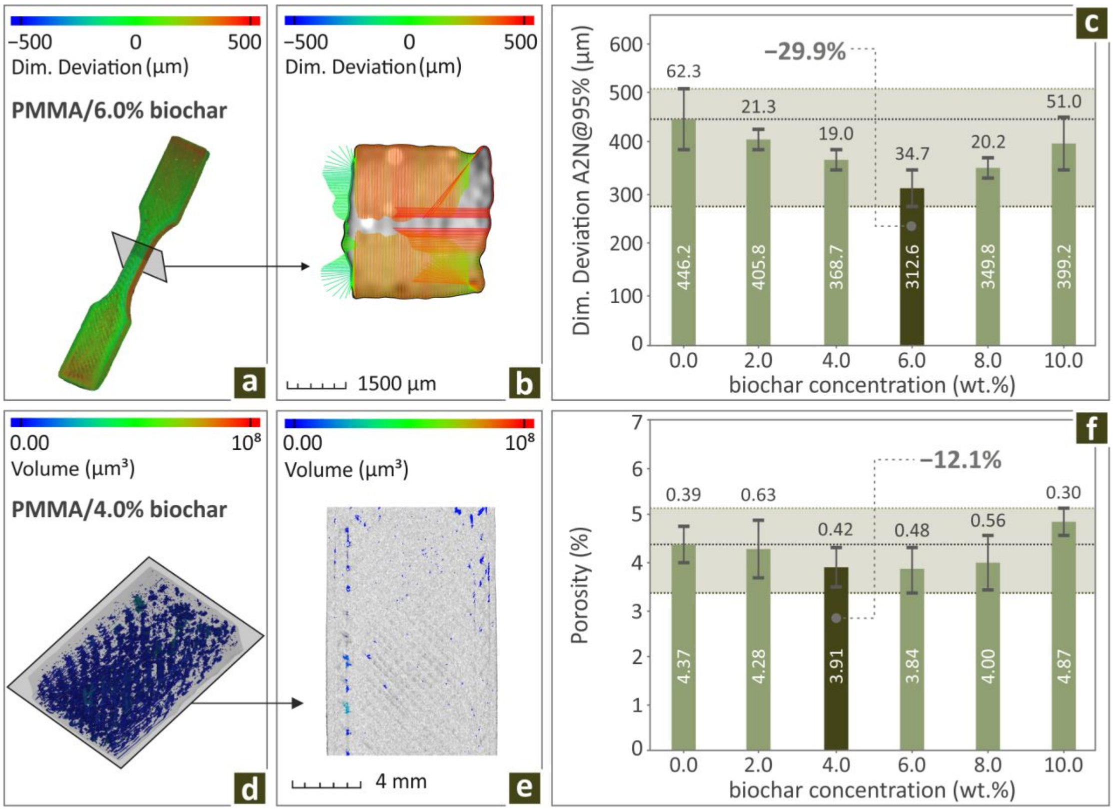

Figure S6. Structural characteristics of PMMA/ Biochar 3D printed samples namely (a-b) dimensional deviation of a PMMA/ Biochar 6.0 wt. % specimen by color-coding mapping, (c) dimensional deviation levels in bars of all PMMA/ Biochar composite samples, (d-e) representation of PMMA/ Biochar 4.0 wt. % volume through color-coding mapping, (f) bars of the porosity levels for all of the PMMA/ Biochar composite samples

S.8. Summary of experimental findings

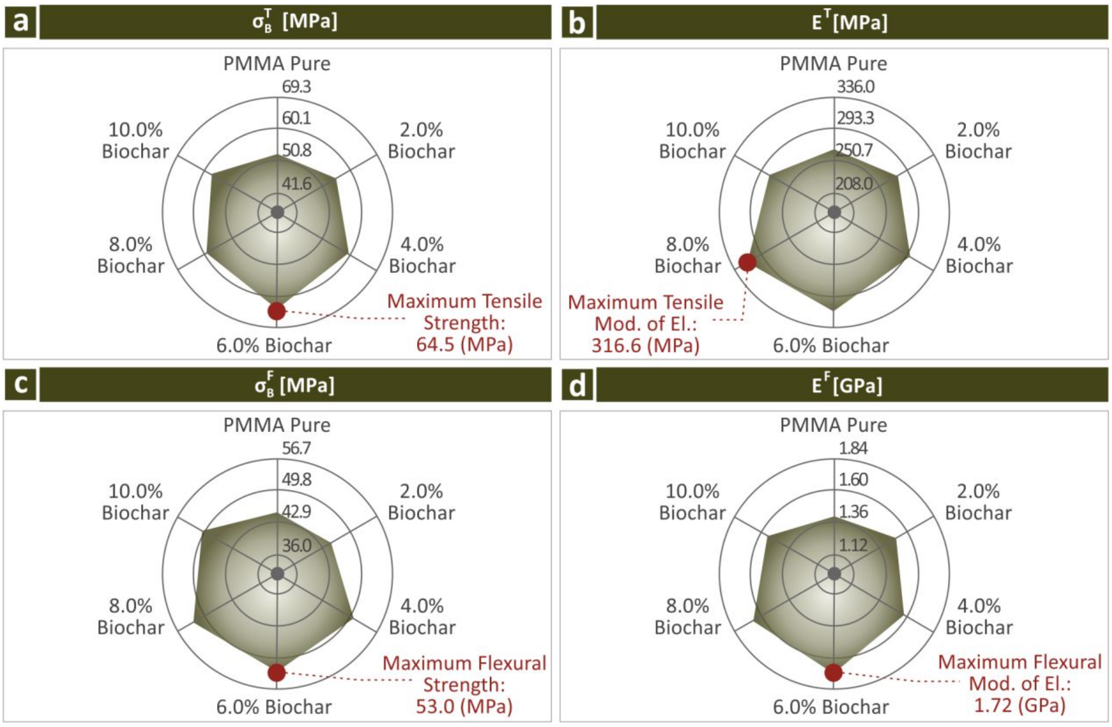

Figure S7. Spider-shaped graphs presenting a summarization of (a) tensile strength, (b) tensile modulus of elasticity, (c) flexural strength, and (d) flexural modulus of elasticity, for all of the PMMA/ Biochar composite samples

References

(1) Veluthandath, A. V.; Bisht, P. B. Identification of Whispering Gallery Mode (WGM) Coupled Photoluminescence and Raman Modes in Complex Spectra of MoS 2 in Polymethyl Methacrylate (PMMA) Microspheres. *J Lumin* 2017, *187*, 255–259. https://doi.org/10.1016/j.jlumin.2017.03.031.

(2) Zimmerer, C.; Matulaitiene, I.; Niaura, G.; Reuter, U.; Janke, A.; Boldt, R.; Sablinskas, V.; Steiner, G. Nondestructive Characterization of the Polycarbonate - Octadecylamine Interface by Surface Enhanced Raman Spectroscopy. *Polym Test* 2019, *73*, 152–158. https://doi.org/10.1016/j.polymertesting.2018.11.023.

(3) Stuart, B. H. Temperature Studies of Polycarbonate Using Fourier Transform Raman Spectroscopy. *Polymer Bulletin* 1996, *36* (3), 341–346. https://doi.org/10.1007/BF00319235.

(4) Resta, V.; Quarta, G.; Lomascolo, M.; Maruccio, L.; Calcagnile, L. Raman and Photoluminescence Spectroscopy of Polycarbonate Matrices Irradiated with Different Energy 28Si+ Ions. *Vacuum* 2015, *116*, 82–89. https://doi.org/10.1016/j.vacuum.2015.03.005.

(5) Makarem, M.; Lee, C. M.; Kafle, K.; Huang, S.; Chae, I.; Yang, H.; Kubicki, J. D.; Kim, S. H. Probing Cellulose Structures with Vibrational Spectroscopy. *Cellulose* 2019, *26* (1), 35–79. https://doi.org/10.1007/s10570-018-2199-z.

(6) Lin, Z.; Guo, X.; He, Z.; Liang, X.; Wang, M.; Jin, G. Thermal Degradation Kinetics Study of Molten Polylactide Based on Raman Spectroscopy. *Polym Eng Sci* 2021, *61* (1), 201–210. https://doi.org/10.1002/pen.25568.

(7) Badr, Y. A.; Abd El-Kader, K. M.; Khafagy, R. M. Raman Spectroscopic Study of CdS, PVA Composite Films. *J Appl Polym Sci* 2004, *92* (3), 1984–1992. https://doi.org/10.1002/app.20017.

(8) Hu, C.; Chen, X.; Chen, J.; Zhang, W.; Zhang, M. Q. Observation of Mutual Diffusion of Macromolecules in PS/PMMA Binary Films by Confocal Raman Microscopy. *Soft Matter* 2012, *8* (17), 4780–4787. https://doi.org/10.1039/C2SM07299H.
